# Chitosan-Based Nanoparticles for Targeted Nasal Galantamine Delivery as a Promising Tool in Alzheimer’s Disease Therapy

**DOI:** 10.3390/pharmaceutics15030829

**Published:** 2023-03-03

**Authors:** Dilyana Georgieva, Denitsa Nikolova, Elena Vassileva, Bistra Kostova

**Affiliations:** 1 Department of Pharmaceutical Technology and Biopharmacy, Faculty of Pharmacy, Medical University of Sofia, 2 Dunav Str., 1000 Sofia, Bulgaria; 2 Laboratory on Structure and Properties of Polymers, Faculty of Chemistry and Pharmacy, University of Sofia, 1 James Bourchier Blvd., 1164 Sofia, Bulgaria

**Keywords:** galantamine hydrobromide, chitosan, alginate, nanoparticles, Alzheimer’s disease therapy, intranasal delivery

## Abstract

Natural alkaloid galantamine is widely used for the treatment of mild to moderate Alzheimer’s dementia. Galantamine hydrobromide (GH) is available as fast-release tablets, extended-release capsules, and oral solutions. However, its oral delivery can cause some unwanted side effects, such as gastrointestinal disturbances, nausea, and vomiting. Intranasal administration is one possible way to avoid such unwanted effects. In this work, chitosan-based nanoparticles (NPs) were studied as potential GH delivery vehicles for nasal application. The NPs were synthesized via ionic gelation and studied using dynamic light scattering (DLS) as well as by spectroscopic and thermal methods. The GH-loaded chitosan–alginate complex particles were also prepared as a way to modify the release of GH. The high loading efficiency of the GH was confirmed for both types of particles, at 67% for the GH-loaded chitosan NPs and 70% for the complex chitosan/alginate GH-loaded particles. The mean particle size of the GH-loaded chitosan NPs was about 240 nm, while the sodium alginate coated chitosan particles loaded with GH were expectedly bigger, with a mean particle size of ~286 nm. GH release profiles in PBS at 37 °C were obtained for both types of NPs, and it was found that the GH-loaded chitosan NPs allowed the prolonged release of the incorporated drug for a period of 8 h, while the complex GH-loaded chitosan/alginate NPs released the incorporated GH faster. The stability of the prepared GH-loaded NPs was also demonstrated after 1 year of storage at 5 °C ± 3 °C.

## 1. Introduction

Alzheimer’s disease is the most common type of dementia. This neurological condition is caused by neuronal degeneration, which leads to memory loss and cognitive decline. The condition usually affects people aged 65 years and over [1]. The treatment of Alzheimer’s disease is only symptomatic with substances, which reduce the symptoms and help improve quality of life [2]. Drugs called cholinesterase inhibitors are used to ease cognitive symptoms, including memory loss, confusion, altered thought processes, and judgment problems. They improve neural communication across the brain and slow the progress of these symptoms.

Galantamine hydrobromide (GH) is one of the drugs used for the treatment of mild to moderate Alzheimer’s dementia approved by the Food and Drug Administration (FDA). It is a reversible competitive inhibitor of cholinesterase, the enzyme responsible for inactivating the neurotransmitter acetylcholine. GH prevents the hydrolysis of acetylcholine into inactive molecules, meaning it can perform its cognitive functions in the brain [3]. Kumar et al. and Suh et al. reported that GH and its derivatives could provide effective treatment for behavioral, functional, and cognitive symptoms of Alzheimer’s disease [4]. Galantamine exhibits favorable pharmacokinetic characteristics, i.e., predictable linear elimination kinetics at the recommended maintenance doses (16 and 24 mg/day), a relatively short half-life (approximately 7 h), and high bioavailability. It is metabolized mainly in the liver and has a low potential for clinically significant drug–drug interactions [5]. GH is delivered orally as a solution, tablets, and capsules. However, its oral delivery can cause some unwanted side effects, such as gastrointestinal disturbances, nausea, and vomiting [6]. These side effects can be avoided via intranasal administration. Apart from this, the nasal route represents a simple and non-invasive way to entirely bypass the blood–brain barrier [7] and to achieve direct drug delivery to the brain [8,9] and less systemic exposure.

Chitosan is a polymer studied intensively for the intranasal delivery of different drug substances, such as vaccines [10], hesperidine [11], carbamazepine [12], insulin [13], and galantamine [14,15,16,17]. It is a polysaccharide composed of D-glucosamine and N-acetyl-D-glucosamine units, obtained via the deacetylation processing of chitin, which is the second most abundant polysaccharide in nature after cellulose [18,19]. The physicomechanical properties (solubility, toxicity, hydrophobicity) of chitosan depend on its degree of deacetylation and molecular weight [20]. The increasing interest in chitosan for biomedical applications is due to its advantageous properties, namely its biocompatibility, low toxicity, low immunogenicity, and antibacterial activity. The good biocompatibility of chitosan is due to its structural and functional resemblance to glycosaminoglycans in the extracellular matrix of the human body and its easy degradation by lysozyme, chitinases, and colon-residing bacteria [21]. The antibacterial activity is due to the presence of positively charged amine groups, which can interact with the negatively charged components of the bacterial cell wall. The cationic group in chitosan molecules also contributes to its mucoadhesive properties, because this group can interact with the anionic acids in the mucous [22]. The low toxicity and low immunogenicity of chitosan using different routes of administration were studied and proven by a number of scientists [23]. Chitosan is also characterized by its high versatility and flexibility for surface modifications, which make it suitable for the preparation of fibers, gels, films, sponges, nanoparticles, scaffolds, and membranes [24,25]. Chitosan-based nanoparticles are biodegradable but remain stable vehicles for drug delivery to the central nervous system, and they are preferred particularly for the delivery of medications used in the treatment of Alzheimer’s disease [26,27,28]. The cationic nature of chitosan opens tight junctions of the blood–brain barrier, allowing drugs to enter the brain [29]. Additionally, chitosan nanoparticles can be easily obtained via the ionic gelation method using tripolyphosphate pentasodium (TPP) as precipitating agent [30]. In spite of its advantages, when chitosan is used alone as a drug carrier, it has poor mechanical properties when wetted and low solubility at pH > 6.5 [31]. This requires the employment of different strategies in order to overcome these problems, which can be achieved by its combination with other materials [32].

One of these strategies is the complexation of chitosan with alginate, which is performed in relatively mild conditions without the application of organic solvents. Alginate is one of the most studied materials for complexation with chitosan. The most important characteristic of alginate is the presence of carboxylic acid groups of mannuronic and guluronic acid units, which when deprotonated could interact electrostatically with the positively charged amino groups of the chitosan-forming polyelectrolyte complex [33]. This complex is biodegradable and biocompatible, and with good mechanical properties at low pH values at which chitosan is soluble [34].

To our best knowledge, GH has been incorporated only into chitosan [15,16,17] and thiolated chitosan particles [14], but not into complex chitosan/alginate particles. Therefore, the aim of the present study was to prepare GH-loaded chitosan and complex chitosan/alginate nanoparticles to compare their main characteristics and to investigate the influence of the alginate shell upon the drug release rate and the mechanism of the release process. The prepared particles will be subjected to further in vivo investigations for the intranasal delivery of GH.

## 2. Materials and Methods

### 2.1. Materials

The GH was supplied by Sopharma AD (Sofia, Bulgaria). The chitosan, TPP, and alginic acid were purchased from Sigma-Aldrich (St. Louis, MO, USA). The glacial acetic acid was obtained by Labimex Ltd. (Sofia, Bulgaria). 

### 2.2. Methods

#### 2.2.1. Preparation of Chitosan and Complex Chitosan/Alginate NPs Loaded with GH

The particles were prepared according to the method described by Calvo et al. [35]. A solution of chitosan (1.25 mg/mL) was prepared by dissolving chitosan in a 1% (*v*/*v*) acetic acid solution using an electromagnetic stirrer for 24 h. The pH of the solution was adjusted to 5.5. The TPP was dissolved in deionized water (0.75 mg/mL) and the resulting solution was added to the chitosan solution dropwise at room temperature.

The GH-loaded chitosan particles were prepared as follows. Chitosan (1.25 mg/mL) and GH (0.35 mg/mL) were dissolved in a 1% (*v*/*v*) acetic acid solution under intense stirring for 24 h. A solution of TPP (0.75 mg/mL) was added dropwise with vigorous stirring for 30 min.

Some of the obtained chitosan particles and GH-loaded chitosan particles were used to prepare complex chitosan/alginate particles according the following procedure. A solution of the previously obtained chitosan particles or GH-loaded chitosan particles was added to a solution of sodium alginate (2 mg/mL). The resulting solution was left overnight at room temperature until the complete formation of the particles was achieved, and then stored in a refrigerator.

#### 2.2.2. Loading Efficiency (%)

The loading efficiency (LE%) was determined by centrifugation. The GH-loaded NPs were separated from the solution by ultracentrifugation (Beckman Optima^TM^ LE-80 K Ultracentrifuge, GMI, Ramsey, MN, USA) at 14,000 rpm for 40 min. The supernatants that had recovered from centrifugation were decanted. The amount of non-incorporated GH in the supernatant was determined at 288 nm using a Hewlett-Packard 8452 A Diode Array spectrophotometer (Walldorf, Germany). The samples were prepared and measured in triplicate. The LE (%) was calculated using the following equation:

$$\;\mathrm{LE}\%=\frac{\mathrm{total}\;\mathrm{amount}\;\mathrm{of}\;\mathrm{GH}\left(g\right)-\;\mathrm{free}\;\mathrm{amount}\;\mathrm{of}\;\mathrm{GH}\left(g\right)}{\mathrm{total}\;\mathrm{amount}\;\mathrm{of}\;\mathrm{GH}\left(g\right)}\times 100$$

#### 2.2.3. Dynamic Light Scattering and Z-Potential Measurements

The obtained solutions were diluted and subjected to a DLS analysis with a Zetasizer Nano ZS apparatus (Malvern Instruments, Worcestershire, UK). The device consists of a 632 nm HeNe gas laser and an optical detector equipped with an ITT FW 130 photo magnifier. The particle size distribution and zeta potential were determined by measuring the scattering of the incident beam at an angle of 173°. Three measurements were made for each sample at 25 °C.

#### 2.2.4. Transmission Electron Microscopy (TEM)

Chitosan/alginate particles and GH-loaded chitosan/alginate particles were studied by TEM. To this end, 100 µL of each solution was dropped on a copper gird. The grids were placed and examined by TEM (JEOL-2100, 200 kV, JEOL Ltd., Tokyo, Japan).

#### 2.2.5. Attenuated Total Reflectance Infrared Spectroscopy (ATR-IR)

Freeze-dried samples of chitosan particles, GH-loaded chitosan particles, chitosan/alginate particles, GH-loaded chitosan/alginate particles, and pure GH were characterized via infrared spectroscopy (IR) in the regime of attenuated total reflectance by using an IRAffinity-1 Shimadzu Flourier Transform Infrared (FTIR) spectrophotometer with a Miracle Attenuated Total Reflectance Attachment. The samples were studied without a preliminary preparation stage.

#### 2.2.6. Diffraction Scanning Calorimetry (DSC)

DSC measurements were performed in order to characterize the thermal behavior of the individual polymers, pure drug, and GH-loaded particles. For the analysis apparatus, Q200 TA Instruments, New Castle, DE, USA, was used. The samples were heated from −30 °C to 300 °C with a heating rate of 10 °C per minute under a nitrogen flow of 50 mL/min.

#### 2.2.7. In Vitro Drug Release Study

The study was performed using a water shaking bath (IKASH-B20, Staufen, Germany). The tests were performed at a rotation speed of 50 rpm and maintained at 37 ± 0.5 °C in 100 mL PBS (pH 7.2). Five milliliter samples of the prepared solutions were placed in dialysis membranes (MWCO 500–1000 Da). At certain intervals, aliquots (2 mL) were withdrawn for the analysis. After each sampling stage, the volume was restored with 2 mL of PBS. The amount of GH released was determined by UV spectroscopy (absorbance at 288 nm) using a Hewlett-Packard 8452 A Diode Array spectrophotometer. Each in vitro drug release experiment was performed in triplicate. The percentage of drug release was calculated using the data obtained from the study.

#### 2.2.8. Drug Release Kinetics

In order to predict the drug release profiles, four kinetic models including zero-order, First-order, Higuchi, and Korsmeyer–Peppas models were applied.

- Zero-order model—this model is used to describe occasions when the drug release rate is not dependent on its concentration. This means that the drug concentration does not affect the release rate:

(1) $$\frac{M\left(t\right)}{M\left(\infty \right)}=k_{0}t$$

- First-order model—this model is used to describe release process when the drug release rate is directly proportional to the drug concentration, i.e., the greater the concentration, faster the release. Because of the proportionality between the rate of reaction and the drug concentration, the first-order process follows linear kinetics. The first-order process is also called the monoexponential rate process and is characterized by logarithmic or exponential kinetics, i.e., a constant fraction of drug is released per unit time:

(2) $$\frac{M\left(t\right)}{M\left(\infty \right)}=e^{-k_{1\;}t}$$

- Higuchi model—according to this model, there are two mechanisms responsible for controlling the drug release rate: swelling and erosion or degradation. These mechanisms lead to the formation of a layer upon the surface of the drug and prevent the entry of an additional amount of water, which results in decreased drug release over time. In the Higuchi model, *k_H_* is a constant proportional to the burst release rate of the release process:

(3) $$\frac{M\left(t\right)}{M\left(\infty \right)}=k_{H}t^{\frac{1}{2}}$$

- Korsmeyer–Peppas model—this model is used to describe the drug release process from polymeric NPs or when the release follows several kinetics mechanisms. In the Korsmeyer–Peppas model, the constant k_KP_ depends on the characteristics of the system, and the coefficient n indicates the nature of the release mechanism. When *n* ≤ 0.5, the release is dominated by the Fickian diffusion mechanism; in the case when 0.5 ≤ *n* ≤ 1, the release follows the mechanism of an abnormal diffusion (non-Fickian diffusion), and when *n* > 1, the release is based on a complex transport mechanism (super-case-II transport):

(4) $$\frac{M\left(t\right)}{M\left(\infty \right)}=k_{KP}t^{n}$$

In the equations, *M*(*t*) represents the amount of GH released at time *t* and *M*(∞) represents the total amount of GH loaded in the NPs; k_0_, k_1_, k_H_, and k_KP_ are the constants of the zero-order, first-order, Higuchi, and Korsmeyer–Peppas models.

#### 2.2.9. Storage Stability

Some of the prepared samples were stored for a period of 1 year in a refrigerator (5 °C ± 3 °C) in order to assess their stability. The particle size and Z-potential were measured at predetermined time intervals (3, 6, 9, and 12 months) according to the procedures described above.

## 3. Results and Discussion

### 3.1. Preparation of Chitosan and Complex Chitosan/Alginate NPs Loaded with GH

Since chitosan has a pKa of 6.5, it is insoluble in water but soluble in acidic solutions. Therefore, a weak acid, namely acetic acid, was used as a solvent to dissolve the chitosan [36]. The chitosan NPs were prepared using the ionotropic gelation method and TPP as the crosslinking agent. Chitosan undergoes gelation due to the complexation with oppositely charged TPP, which leads to the formation of spherical particles. The method does not apply any harmful organic solvents and it is conducted at room temperature, and at the same time can efficiently preserve the bioactivity of the drug during encapsulation. The mechanism of the particle formation is based on the electrostatic interaction between the amine group of the chitosan and the negatively charged group of the polyanion TPP. This method offers mild preparation conditions in an aqueous medium. The procedure can be divided into two stages—the preparation of the GH-loaded chitosan NPs and the coating of the obtained particles with sodium alginate in order to modify the release of GH from the NPs.

### 3.2. Loading Efficiency (%)

The LE (%) was determined by centrifugation. The results are presented in Table 1. High LE (%) values were observed for both types of particles, with 67% for the GH-loaded chitosan NPs and 70% for the complex chitosan/alginate GH-loaded particles. This high LE proved the suitability of the preparation method that was used.

### 3.3. Particle Size and Z-Potential

The particle size and Z-potential were evaluated by DLS. The results are presented in Table 1. The average size of the unloaded chitosan NPs was about 215 nm and that of the chitosan/alginate NPs was about 97 nm. From the results, it can be seen that the unloaded complex particles were smaller than the chitosan particles. This phenomenon has been noted and discussed by other scientific groups [37,38]. They also found that the addition of alginate led to the formation of more compact particles with smaller sizes. This could be explained by the pH of the alginate solution, which was in the range of 5.3–6.0, allowing a stronger interaction between the chitosan and alginate. At this pH, the majority of the amine groups of the chitosan were protonated and were able to participate in electronic interactions with the carboxyl group of the alginate.

From Table 1, it can be seen that the mean particle size of the prepared GH-loaded chitosan NPs was about 240 nm. The coating of the particles with sodium alginate led to the formation of bigger particles with a mean particle size of about 286 nm. This was probably due to the presence of GH, which limits the interaction between the chitosan and alginate. 

When TPP was added to the chitosan solution, complexes were spontaneously formed, which had an overall positive surface charge, as confirmed by the Z-potential measurements. As can be seen in Table 1, the Z-potential values for chitosan NPs and GH-loaded chitosan NPs were 67 mV and 55.3 mV, respectively. These results showed a slight decrease in the Z-potential after the addition of GH. The Z-potential established for the complex chitosan/alginate particles was −36.6 mV. Generally, values more electronegative than −30 mV lead to an increase in the stability of the system. The excess of alginate is necessary for the efficient electrostatic stabilization of the NPs against agglomeration due to the efficient negative surface charge. The negative charge of the NPs is also appropriate in order to avoid unwanted non-specific interactions with body tissues, membranes, and proteins.

### 3.4. Transmission Electron Microscopy (TEM)

**Figure 1:**
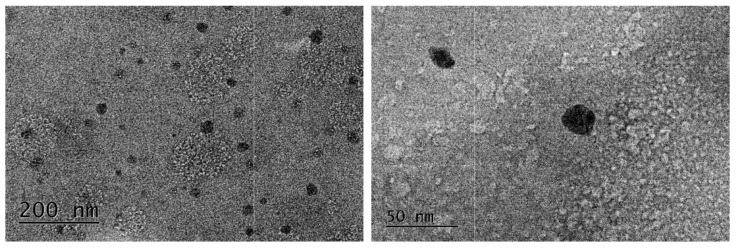
TEM images of chitosan/alginate NPs at different magnifications.

The obtained TEM images of the chitosan/alginate NPs are presented in Figure 1. The non-loaded chitosan/alginate NPs exhibited an almost spherical shape and a small size range of about 20–30 nm.

**Figure 2:**
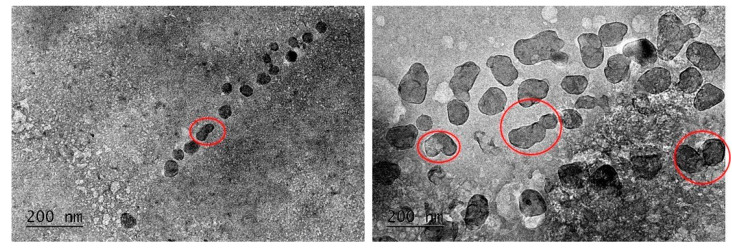
TEM images of GH-loaded chitosan/alginate NPs at different magnification.

From the images presented in Figure 2, it is obvious that the GH-loaded chitosan/alginate particles were characterized by their irregular shape and bigger size range of about 50–100 nm. This could be explained by the drug loading, which causes the enlargement of the NPs due to the drug’s inclusion between the polymer chains and the expansion of the polyelectrolyte complexes. Coalescence of two or more particles was also observed (see the red circles in Figure 2), which could be explained by the slightly reduced Z-potential of the drug–polymer complex (Table 1). This result corresponds with the results obtained by DLS, where GH-loaded particles with bigger sizes were compared to the non-loaded ones.

When comparing the particle sizes obtained using both DLS and TEM, significant differences in the results can be seen. Such discrepancies have been noted and discussed by other scientific groups. This is presumably due to the fact that samples for TEM imaging are prepared by drying, while DLS determines the hydrodynamic diameter, which includes the hydrophilic layers surrounding the particles in the solution as well as the swelling of the polymer particles in the aqueous solution [39,40].

### 3.5. Attenuated Total Reflectance Infrared Spectroscopy (ATR-IR)

**Figure 3:**
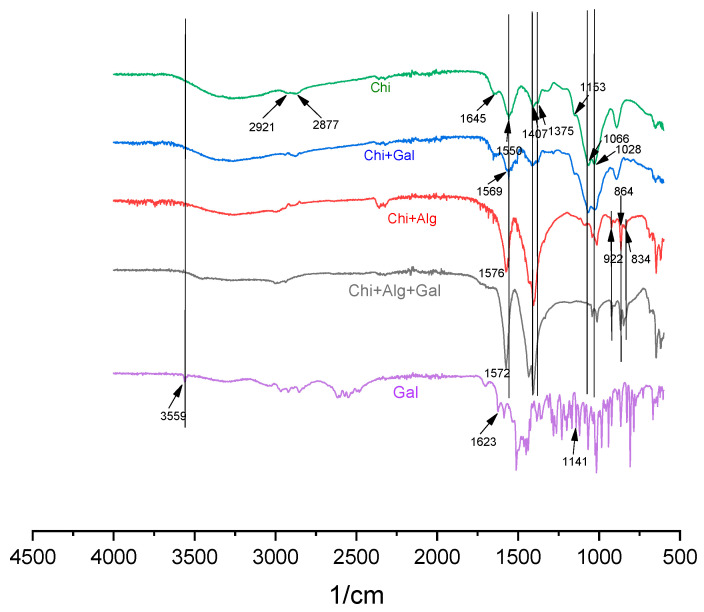
ATR-IR spectra of chitosan, chitosan/alginate complexes, GH-loaded chitosan particles, GH-loaded chitosan/alginate particles, and pure GH.

The results obtained from the ATR-IR experiments are shown in Figure 3. It can be seen that all characteristic bands of chitosan are observed in the spectra: the broad band in the region of 3600–3290 cm^−1^ is due to N-H, O-H, and intramolecular hydrogen bond stretching. The bands at 2921 cm^−1^ and 2877 cm^−1^ are characteristic of a large number of polysaccharides and are due to symmetric and non-symmetric oscillations of C-H bonds. The band at 1645 cm^−1^ can be attributed to the N-acetyl groups’ residuals, more specifically to the C=O stretching of amide I. This band is almost invisible in the other three spectra, which can be attributed to the interactions occurring between the chitosan and other compounds. The band at 1550 cm^−1^ is characteristic of N-H bending of amide II. This band can be observed also in the spectra of GH-loaded chitosan particles. In the spectra of chitosan/alginate and GH-loaded chitosan/alginate particles, this band is shifted. In the spectra of the former, the band is shifted to 1576 cm^−1^, which can be attributed to the interactions between the oppositely charged chitosan and alginate macromolecules. On the other hand, the shifting of the same band in the spectra of the GH-loaded chitosan/alginate particles could be associated with the interactions between the drug and the polymers.

Other characteristic bands for chitosan that can be seen in the spectra of pure and loaded polymers but are shifted in the spectra of complexes are those at 1407 cm^−1^, 1375 cm^−1^, 1066 cm^−1^, and 1028 cm^−1^. These bands are characteristic of the oscillations and stretching of CH_2_, CH_3_, and C-O, respectively. 

The most characteristic bands of alginate are at 946 cm^−1^, corresponding to the uronic acid residuals, while those at 883 cm^−1^ and 810 cm^−1^ correspond to the oscillations and stretching of the bonds of mannuronic acid residuals. All bands are shifted in the spectra of chitosan/alginate particles and GH-loaded chitosan/alginate particles at 922 cm^−1^, 864 cm^−1^, and 834 cm^−1^, respectively. Most probably this effect is due to the interactions between both polymers in the complex.

From the spectra of pure GH, it can be concluded that it is a crystalline substance. The most specific band for the drug is at 3559 cm^−1^. This band is absolutely overlapped in the other two spectra, namely for GH-loaded chitosan particles and GH-loaded chitosan/alginate particles. This observation is evidence that the drug is incorporated in the polymers and is amorphous.

### 3.6. Differential Scanning Calorimetry (DSC)

**Figure 4:**
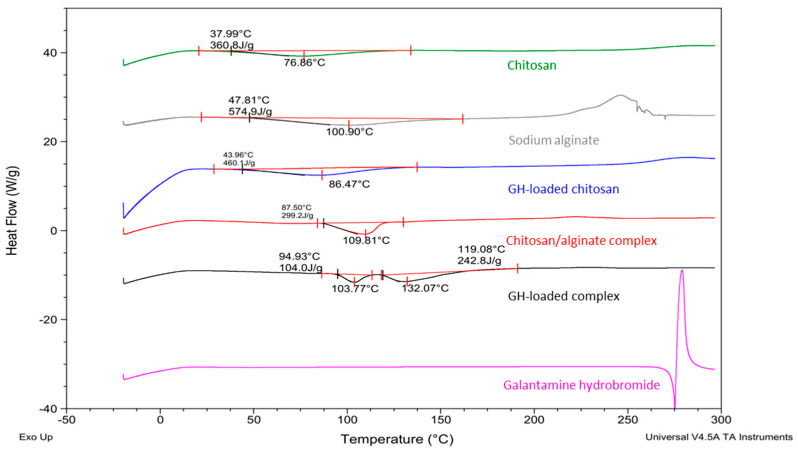
DSC thermograms of chitosan, sodium alginate, GH, GH-loaded samples, and chitosan/alginate complexes.

The thermal behaviors of all samples, as well as of their constituents, namely chitosan, alginate, and GH, were observed via DSC. The thermograms are presented in Figure 4. In all samples’ thermograms, a broad peak around 100 °C was observed. Most likely, this peak was due to water evaporation. Such an evaporation peak was not observed in the drug’s thermogram because it was not hygroscopic. The water content in the samples was calculated, using the rule that the enthalpy of 100% water is 2400 J/g [41]. All results are shown in Table 2. It can be seen that the GH-loaded chitosan particles, as well as the GH-loaded chitosan/alginate complexes, had a lower water content compared to the non-loaded NPs. This effect could be attributed to the incorporation of the drug substance, which is not hygroscopic, and on the other hand its interactions with the polymers replaces water molecules, thereby decreasing the sample’s moisture content.

In the GH thermogram, a melting peak at around 270 °C was observed confirming the crystallinity of the pure drug, as already evidenced by the ATR-IR spectrum (Figure 3, Table 2). This peak was not seen in the thermogram of GH-loaded chitosan particles, which was an indication of drug amorphization when loaded into the chitosan. This observation is in line with the ATR-IR data. On the other hand, in the GH-loaded chitosan/alginate particle thermogram, the peak corresponding to the melting point of the GH was shifted to ~130 °C. This value is very close to the melting point of pure GH, which according to the literature is approximately 126 °C [42]. Thus, most probably, during the formation of GH-loaded chitosan NPs and chitosan/alginate particles, the drug was first dissolved in chitosan aqueous solution, in which it dissociated into Br^−^ anions and a GH cation. Upon the particles’ formation, pure galantamine (not its salt) was incorporated into the particles, as demonstrated by the lower melting temperature (Table 2).

### 3.7. In Vitro Drug Release

**Figure 5:**
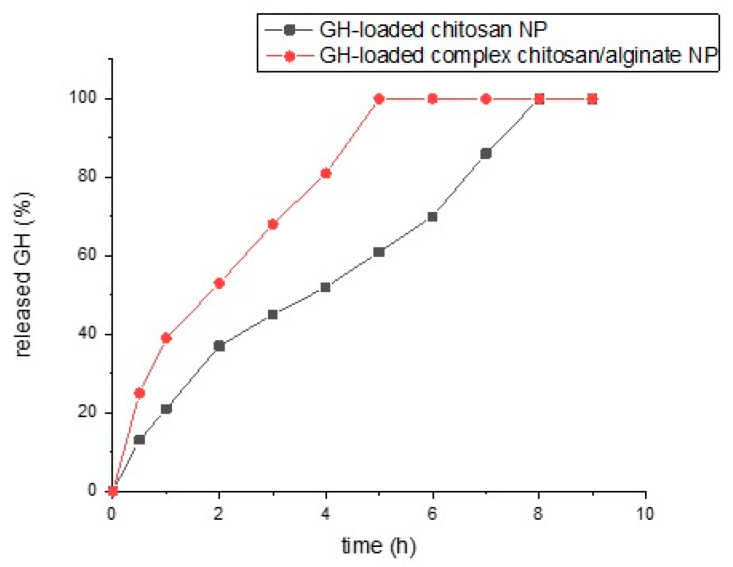
GH release from chitosan NPs and complex chitosan/alginate NPs in PBS (pH 7.2) at 37 °C.

The ability of chitosan to form ionic crosslinks leads to the formation of stable complexes that release the drug over a prolonged period of time, thereby achieving controlled drug release [43]. This could be especially beneficial for drugs that are water-soluble, such as GH. Therefore, it could be expected that the incorporation of GH into chitosan NPs would achieve a delay in the drug release rate. From the results obtained from the in vitro GH release study presented in Figure 5, it can be seen that both types of particles managed to delay the release of the water-soluble GH to different extents. No significant burst release was observed, which indicated that the drug was homogeneously dispersed in the polymer matrix. As was expected, the GH-loaded chitosan NPs provided prolonged the release of the incorporated drug for a period of 8 h. The delay in the release of the highly soluble GH was due to its incorporation into the chitosan particles, which were sparingly soluble at pH 7.2 in the medium, where the study was performed [44]. From the results shown in Figure 5, it is obvious that the complex GH-loaded chitosan/alginate NPs released the incorporated GH faster than the chitosan NPs. This may have been due to the fact that at pH 7.2 the alginate shell is highly soluble and cannot protect the drug. From the presented results, it can be concluded that the GH-loaded chitosan particles were more suitable for achieving the prolonged release of GH compared to the complex chitosan/alginate particles at neutral pH.

### 3.8. Drug Release Kinetics

To study the drug release profiles, different mathematical models were applied. The correlation coefficient (R^2^) was used in order to compare the different models, whereby a value closer to 1 indicated a better correlation. In Table 3, the correlation coefficients (R^2^), the constants of the different models, and the release exponent (n) derived after fitting the experimental data of GH release from the both types of NPs are listed. For GH-loaded chitosan NPs, it was observed that the GH release from the samples was best described by the Higuchi model (R^2^ > 0.95). The drug release from the complex particles was found to be best fitted again by the Higuchi square root model (R^2^ = 0.99). These results indicated that the drug release from the particles was a diffusion-controlled process. Additionally, from the Higuchi model, the values of k_H_ for both types of NPs were very low (4.4010 and 5.5785), which indicated a negligible burst release. This was also proven by the drug release studies.

Once it was established that the prime mechanism of the drug release was diffusion, the type of diffusion needed to be specified. In order to do this, the release data were fitted using the empirical equation proposed by Korsmeyer and Peppas. According to this model, the type of diffusion could be evaluated from the value of the release exponent (n). From the results presented in Table 3, it is obvious that the release follows the mechanism of abnormal diffusion (non-Fickian diffusion) (*n* ≥ 0.5). This means that due to polymer swelling and eventual particle erosion (polymer chains start to slowly dissolve in the release medium and the particles homogeneously erode), the drug diffusion becomes controlled by these two simultaneously running processes, which results in the observed non-Fickian drug diffusion [45].

### 3.9. Storage Stability

In order to establish the suitability of the obtained particles as drug delivery systems, their stability was investigated, as this directly affects their efficacy. For this purpose, the changes in particle size and Z-potential were investigated. Based on the literature data for stability studies of chitosan NPs conducted by other scientific groups, chitosan NPs should not be stored at room temperature, as they are prone to degradation compared to those stored in cool environments. This may be due to the fact that at low temperatures the kinetic motion of the particles is slow and aggregation is less likely to occur, meaning the particles can maintain their spherical shape [46]. According to these data, we expected that there would be no significant change in stability under the storage conditions (5 °C ± 3 °C). The particle size and Z-potential were measured at predetermined time intervals (3, 6, 9, and 12 months). Table 1 presents only the results obtained from the measurements carried out for the 12th month, because there was no significant difference compared to the results obtained for the 3rd, 6th, and 9th months. As can be seen from the data, the size and Z-potential of the prepared NPs remained almost unchanged during the storage period, which was evidence of their stability.

## 4. Conclusions

In the present work, chitosan-based NPs were studied for potential nasal GH delivery. The NPs were synthesized using the ionic gelation method and the GH was loaded during the particle preparation process. Some of the obtained GH-loaded chitosan particles were coated with alginate via chitosan–alginate complexation in order to investigate the influence of the alginate layer upon the drug release process.

High LE (%) values were observed for both types of particles, with 67% for the GH-loaded chitosan NPs and 70% for the complex chitosan/alginate GH-loaded particles, which proved the suitability of the preparation method used.

The chemical composition of the obtained particles was proved vi ATR-IR spectroscopy and DSC. Their nanosized structure was observed by TEM and confirmed by DLS. The mean particle size of the prepared GH-loaded chitosan NPs was about 240 nm. The coating of the particles with sodium alginate led to the formation of bigger particles with a mean particle size of about 286 nm. The Z-potential values proved their stability.

The results obtained from the in vitro drug release studies showed that the GH-loaded chitosan NPs allowed the prolonged release of the incorporated drug for a period of 8 h due to the fact that chitosan is hardly soluble at pH 7.2. The complex GH-loaded chitosan/alginate NPs released the incorporated GH faster than the chitosan NPs due to the fact that at pH 7.2 the alginate shell is highly soluble and cannot protect the drug. From the presented results, it can be concluded that the GH-loaded chitosan particles were more suitable for achieving the prolonged release of GH compared to the complex chitosan/alginate particles.

The drug release kinetics were studied and we established that the GH release from the samples was best described by the Higuchi model, which indicated that the drug release from the particles was a diffusion-controlled process.

The stability study showed that the size and Z-potential of the prepared NPs remained almost unchanged during the storage period, making them suitable as drug delivery systems.

## 5. Limitations and Future Research

Due to its unique and versatile biological properties, chitosan has been considered a useful polymer in the pharmaceutical area. Given the wide diversity of chitosan derivatives, choosing the most suitable one can be a problem because the choice depends on the molecular weight, degree of acetylation, and purity grade. The most frequent impurities present in chitosan are ash, heavy metals, and proteins. Proteins can cause immunogenicity issues, while high ash and protein contents may hinder chitosan dissolution and cause difficulties in its preparation [47].

The stability of chitosan is also a very important issue to consider when using it as a vehicle for drug delivery. Chitosan is very sensitive to temperature and humidity, which can change its mechanical properties [48] and cause a partial loss of its mucoadhesive properties [49]. For these reasons, chitosan particles must be stored at low temperatures (2–8 °C) in a dry place.

The toxicological profile of chitosan and its derivatives is still under investigation. To date, chitosan has shown little or no toxicity in animal models, and clinical data in healthy human volunteers are lacking.

Our future research will focus on the estimation of the bioavailability of GH in the mouse brain via intranasal drug delivery using the prepared chitosan and complex chitosan/alginate NPs compared to the oral delivery of the solution using a pharmacodynamic study and biochemical estimation of the acetylcholinesterase activity in the mouse brain.

## Figures and Tables

**Table 1:** Average particle size (nm), Z-potential, and LE (%) values of the different samples.

Composition	Freshly Prepared NPs			Stability Study after 1 Year Storage	
	Average Size (nm) ± SD	Z-Potential (mV) ± SD	LE (%) ± SD	Average Size (nm) ± SD	Z-Potential (mV) ± SD
Chitosan NPs	215 ± 0.9	67 ± 1.3	-	228 ± 0.6	70 ± 0.5
Chitosan + GH NPs	240 ± 0.9	55.3 ± 0.4	67 ± 2.5	252 ± 0.8	53.2 ± 2.1
Chitosan + Alg NPs	97 ± 2.5	−36.6 ± 0.6	-	108 ± 1.7	−38.4 ± 1.3
Chitosan + Alg + GH NPs	286 ± 3.1	−35.5 ± 0.7	70 ± 1.9	304 ± 1.5	−39.3 ± 0.8

**Table 2:** Water contents in pure and drug-loaded samples as well as in their constituents.

Sample	Water Content, [%]	Tm, [℃]
Chitosan	15%	-
Alginate	24%	-
Chitosan/alginate complex	19%	-
GH-loaded chitosan NPs	12%	-
GH-loaded complex NPs	4%	130
GH	-	270

**Table 3:** Parameters derived from the different mathematical models describing the drug release kinetics.

Kinetic Model	Chitosan + GH NPs	Chitosan + Alg + GH NPs
Zero-order	k_0_ = 0.0055	k_0_ = 0.0104
	R^2^ = 0.8272	R^2^ = 0.8979
First-order	k_1_ = 0.0026	k_1_ = 0.0063
	R^2^ = 0.8787	R^2^ = 0.9355
Higuchi	k_H_ = 4.4010	k_H_ = 5.5785
	R^2^ = 0.9541	R^2^ = 0.9858
Korsmeyer–Peppas	n = 0.5001	*n* = 0.5748
	R^2^ = 0.8901	R^2^ = 0.9928

## Data Availability

Not applicable.
